# Effects of positive personal and non-personal autobiographical stimuli on emotional regulation in older adults

**DOI:** 10.1007/s40520-019-01147-0

**Published:** 2019-02-25

**Authors:** L. M. Carretero, J. M. Latorre, D. Fernández, T. J. Barry, J. J. Ricarte

**Affiliations:** 1 Psychology Department, School of Medicine, University of Castilla La Mancha, Albacete, Spain; 2 Department of Psychology, The University of Hong Kong, 6th Floor, Jockey Club Tower, Centennial Campus, Pokfulam Road, Hong Kong, Hong Kong; 3 Department of Psychology, The Institute of Psychiatry, Psychology and Neuroscience, King’s College London, London, UK

**Keywords:** Autobiographical memory, Emotion regulation, Older adults, Positive image

## Abstract

People can regulate negative emotional states using personal episodic information stored in memory. However, amongst older adults, assistance in retrieving personal memories might be needed. As such, positive personal images might better facilitate the retrieval of positive personal memories, relative to generic positive images. The present study induced older adults (*N* = 40; *M*_age_ = 76.28) into a negative mood state using a validated film clip (“Dead Man Walking”; Robbins et al. in Dead Man Walking [Cinta Cinematográfica]. PolyGram Filmed Entertainment, Working Title Films, Estados Unidos, 1995). Participants were then shown positive personal images (album photos) or positive non-personal images from the International Affective Picture System (IAPS) and between-group differences in their mood state and their ability to retrieve positive autobiographical memories were measured. Although participants’ moods decreased after the negative mood induction, their mood then recovered after picture cuing regardless of whether images were personal or non-personal. Furthermore, the positive mood evoked by non-personal, but not personal, images was significantly positively associated with self-reported feelings of reliving of the memories evoked by those images. These results suggest that, when pictures from personal life are not available, the selection of images able to generate positive autobiographical memories with a sense of reliving, is a feasible tool for older adult’s emotional regulation.

**Electronic supplementary material:**

The online version of this article (10.1007/s40520-019-01147-0) contains supplementary material, which is available to authorized users.

## Introduction

Memory is a complex construct comprising various systems. Autobiographical memory, one aspect of episodic memory [1], refers to memories for personally experienced events contextualized in space and time [2–4]. Autobiographical memory is typically defined as a hierarchical network ranging from general memories to highly detailed specific memories [5, 6]. It comprises not only the episodic content of events but also the way we felt during these events [7]. These emotions can be re-experienced once a memory for an event is then later retrieved, such that we can use our memories to regulate our emotional states. Healthy participants who have been asked to remember positive autobiographical memories after negative mood inductions have shown improvements in their mood after memory retrieval [8–10]. These mood-reparatory effects are thought to be driven by re-experiencing of personal events after they are cued and retrieved. As such, if a cue evokes the retrieval of a positive and personally meaningful autobiographical memory this may be more likely to evoke a greater sense of nostalgia and reliving, and so have greater mood-reparatory effects, than if less potent retrieval cues are used [11]. The ability to regulate negative mood states with autobiographical memories may be influenced by the ease with which these memories can be accessed, with more accessible memories providing more rapid and potent mood-reparatory effects than less accessible memories. This issue is particularly pertinent amongst older people who have difficulty accessing autobiographical memories and where these problems are associated with elevated depressive symptoms [12]. However, this possibility has yet to be investigated.

When retrieving memories for autobiographical events people engage in one of two methods of retrieval [5]. In particular, people can engage in generative retrieval where retrieval begins at the most general and abstract level of memory where the self-relevance of the cue is first considered and then gradually more detail is retrieved as a memory for a specific and detailed event that occurred at a particular time and place is eventually retrieved. Alternatively, if much of this self-relevant information is already present within a cue (e.g., the name or picture of a friend), people can engage in direct retrieval where a specific event is more immediately accessed [13, 14]. As such, the extent to which a person engages in generative or direct retrieval depends on the proximity of the cue that is used to elicit retrieval to other self-relevant autobiographical information stored in memory. Compared with younger adults, older adults retrieve autobiographical memories with significantly less specificity and detail [12]. Studies in this area typically use standardised sets of adjectives and nouns (e.g., happy, confident) that elicit generative retrieval. It may be that such memory problems are less evident when direct cues are used, than if generative cues are used, such that the details of their memories are more accessible and they are better able to use these memories to regulate any negative affect that they might be experiencing. To ensure that personal memories are retrieved, and to assist in the accessibility of these memories, the current study utilises personal photographs as direct retrieval cues and contrasts this with non-personal images taken from the International Affective Picture System (IAPS) which are more likely to elicit generative retrieval.

Although no study has examined any possible differential effects of generative, compared with direct, retrieval cues on older adults’ abilities to retrieve memories and use them to regulate their moods, several studies have studied the broader beneficial effects of positive autobiographical memory retrieval amongst older adults [6, 15]. Serrano et al. [6] designed a therapy for older adults with depression in which the clinician used concrete, self-relevant cues to elicit direct retrieval of personal and positive memories for specific events. Post-intervention, participants in this group showed decreased depressive symptomatology, lower levels of despair and greater life satisfaction. In a later study utilising a similar intervention, Life Review [3], older participants showed an improvement in depressive symptomatology and an increase in the ability to retrieve specific memories. It is of note that although these interventions rely on positive personal imagery, such images are not always accessible, or there are not always enough images to evoke sufficient numbers of memories across an entire treatment programme. Also, these interventions are typically delivered in a group format where images might be shared across participants within a group and as such images are not often relevant to every participant within a group. It is therefore important to assess whether non-personal images can elicit similar mood-reparatory effects as personal images amongst older adults, so that such images might be integrated into memory therapeutic interventions.

The present investigation therefore tested whether cues which evoke generative retrieval of positive autobiographical memories (e.g, non-self-relevant positive images such as a picture of a child smiling) were less effective than cues which evoke direct retrieval of positive autobiographical memories (e.g., a self-relevant image such as a picture of one’s own child smiling) at helping older adults regulate negative emotions. Participants were induced into a negative mood state using a validated movie clip (“Dead Man Walking”, Robbins et al. [16]). Next, participants were either presented with positive personal autobiographical photographs that they had supplied themselves or they were shown generic positive images from the International Affective Picture System (IAPS, Lang et al. [17]). Direct cues were expected to elicit stronger feelings of reliving and nostalgia, and as a consequence provide greater benefit in the regulation of the negative mood, than if generative cues were presented. Individual differences in reliving were expected to correlate positively with improvements in mood.

## Methods

### Participants

The study was conducted in accordance with the recommendations set out in record no. 06/2016 of the Clinical Research Ethics Committee of the Castilla-La Mancha Health Service. The study protocol was approved by this committee prior to recruitment.

Participants were included in the present study if they were older than 65 years. Participants were excluded if they had severe visual impairment or if they were diagnosed with an intellectual disability or neurodegenerative disorder. All participants gave their written informed consent following an explanation of the study.

Participants were 40 individuals aged between 65 and 92 years (*M* = 76.28, SD = 7.82) who regularly attended workshops at a senior centre in a town in the region of Castilla-La Mancha (Spain). Participants were randomised into two groups depending on the images used to cue their memories. Participants in the personal group (*n* = 19; *M* = 74.21, SD = 7.54, 42.1% men) were shown positive personal autobiographical images that they supplied themselves and participants in the non-personal group (*n* = 21; *M* = 78.14, SD = 7.77, 9.5% men) were exposed to positive images from the IAPS [16]. There was no between-group difference in participants’ age.

### Measures

#### Self-report questionnaires

Individual differences in anxious and depressive symptoms were assessed using the anxiety (7a) and depression (8a) subscales of the Patient-Reported Outcomes Measurement Information System (PROMIS; Cella et al. [18]). Participants were asked about the frequency with which they experienced in the last 7 days each of the symptoms of anxiety and depression in the last 7 days on scales from one (never) to five (always). Higher scores indicate more severe symptoms. Cronbach´s alpha of 0.89 and 0.87 were obtained for anxiety and depression PROMIS subscales, respectively. Mood state was measured using the Positive and Negative Affect scale (PANAS; Watson et al. [19]), in which respondents are presented with a number of words describing typical positive (e.g., excited) and negative (e.g., irritable) feelings. Participants are required to rate each word from one to five based on the extent to which they felt them over the past week, from one (very slightly or not at all) to five (extremely). Cronbach’s alpha for each subscale was good (positive *α* = 0.77; negative *α* = 0.84).

#### Mood induction

A 7-min scene from the film *Dead Man Walking* [16] in which the main character is executed, was used to induce a negative mood. This scene was previously validated by Fernández et al. [20] who concluded that it provides a valid and reliable method for inducing sadness.

Three Self-Assessment Manikins (SAM; Lang [21]) were used to measure individual differences in feelings of pleasantness, arousal, and dominance. Participants were shown nine-point scales in the form of illustrations of manikins that depict scales from pleasant to unpleasant, nervous to calm, and strong to weak, respectively, for each of the subscales.

#### Memory cuing

Participants in the personal group were asked to bring five personal photos that reminded them of happy moments in their lives. The photos showed situations from childhood, family (children, grandchildren, siblings…) and events (weddings, christenings, family meals…). Participants in the non-personal group were not asked for personal photographs and instead were shown a selection of positive autobiographical images from the IAPS database [17]. Specifically, slide number 1710 (puppies), 2058 (baby), 2150 (baby paternity), 2170 (mother) and 5836 (beach) were selected, representing on average of 7.79 of valence, 4.77 of arousal and 6.37 of dominance. These images were selected to represent life moments that are typically considered to be positive such as the birth of a baby, a new pet or a trip to the beach. The participants of both groups were asked to generate a positive memory from each image shown. After participants said they had recalled a memory for each image, they reported their feelings of nostalgia and sense of reliving the generated memory evoked for them. Nostalgia and reliving were scored on three-point scales such that higher scores indicated greater nostalgia and reliving.

### Procedure

Approval from the Research Ethics Committee of the Albacete University Hospital Complex was first obtained. Before the experimental sessions took place, participants were told about the aversive nature of the video clip and informed consent from participants was obtained. Participants completed the self-reported questionnaires and the first set of SAMs prior to the mood-induction clip. After the clip, participants completed the second set of SAMs and then they underwent their group-designated cuing procedure. Changes in mood were again assessed following the cuing procedure with a third set of SAMs.

Due to the disturbing nature of the film clip, participants were permitted to choose how long they watched. The full clip was viewed by 36.6% (15 participants, Personal group = 9), while 46.3% (19 participants, Personal group = 7) viewed more than 4 min. The remaining 17.1% (six participants, personal group = 3) did not get to see 4 min of the video. There were no significant differences in any SAM scores after video clip viewing between those participants who viewed the complete film and those participants who did not, (pleasantness: *t*(38) = 0.16, *p* = .87; arousal: *t*(38) = − 0.42, *p* = .67; dominance: *t*(38) = − 0.55, *p* = .58).

### Statistical analysis

First, participants’ prior mood states were analysed using a *t*-test to compare possible differences between the two groups for each of the self-report questionnaires and the pre-induction SAM. To examine changes in mood states across the induction, we conducted a mixed analysis of variance with group (personal; non-personal) and time (pre-induction; post-induction; post-cuing) as between- and within-subject factors, respectively. This analysis was conducted separately for each of the SAM scores (valence, arousal, and dominance). Finally, *t*-tests contrasted differences on the levels of nostalgia and reliving evoked by the images between groups and Pearson correlations explored associations between post-cuing SAMs and levels of reliving and nostalgia provoked by images within each group.

## Results

### Pre-induction

No significant between-group differences in anxious and depressive symptoms and scores on the PANAS subscales prior to the induction were found (see Table 1). Regarding the pre-induction SAM, although there were no group differences for valence and dominance, significant differences were found between the two groups for levels of arousal and as such arousal was included as a covariate in our subsequent analyses on SAM scores (Table 2).

**Table 1:** Mean values and standard deviations from the self-report questionnaires

	Personal	Non-personal		
	*M* (SD)	*M* (SD)	*df*	*t*/*x*^*2*^
Age	74.21 (7.54)	78.14 (7.77)	38	-1.62
Gender (female/male)	19/9	21/2	1	5.64
Anxiety	1.68 (0.95)	1.67 (0.91)	38	0.60
Depression	0.89 (0.74)	0.85 (0.85)	38	0.15
PANAS positive	3.61 (0.57)	3.21 (0.63)	31	1.92
PANAS negative	1.95 (0.67)	2.16 (0.60)	31	− 0.93

Differences between participants cued by personal and non-personal images in their self-reported symptoms of anxiety and depression, measured using the subscales of the Patient-Reported Outcomes Measurement Information System, as well as their scores on the subscales of the Positive and Negative Affect Scale

**Table 2:** Change in participants’ mood state

	Personal	Non-personal			
	*M (DT)*	*M (DT)*	*t*	*df*	*F*
Pleasantness					
Pre-induction	6.37 (2.19)	6.00 (1.95)	0.56	37	0.09
Post-induction	1.89 (1.37)	1.80 (1.19)	0.33	38	0.71
Post-cuing	8.26 (0.93)	7.60 (1.47)	1.67	38	3.53
Arousal					
Pre-induction	5.84 (2.06)	3.95 (1.75)	3.14**	38	0.29
Post-induction	7.68 (1.49)	8.00 (1.18)	− 0.75	38	2.27
Post-cuing	4.63 (2.24)	4.29 (1.88)	0.53	38	0.64
Dominance					
Pre-induction	6.21 (1.96)	6.43 (1.99)	− 0.35	38	0.22
Post-induction	3.37 (2.09)	3.38 (2.20)	− 0.02	38	0.59
Post-cuing	6.53 (1.47)	7.00 (1.48)	− 1.01	38	0.04

Table showing the scores on SAM [21] for both groups in the three measurement points

**p < .01

### Change in participants’ mood state

See Fig. 1.

**Fig. 1:**
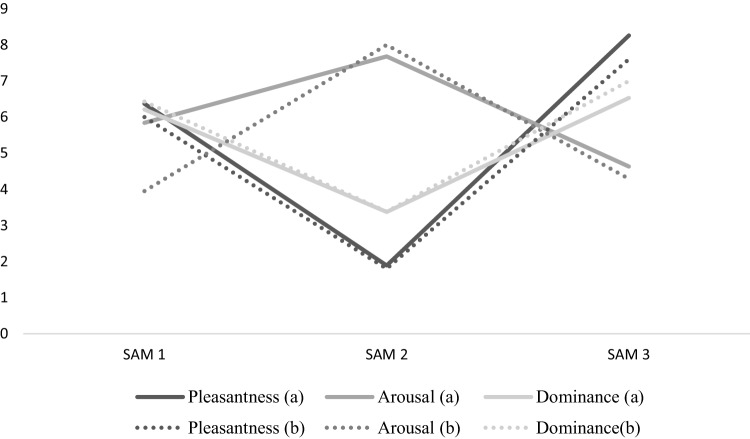
Graph showing scores on SAM [21] for both groups, at the three measurement points. (a) = Positive personal autobiographical images; (b) = positive autobiographical images from the IAPS [17]

#### Pleasantness

The mixed ANOVA for valence ratings showed a significant main effect of time, *F*(2,36) = 261.29, *p* < .001, $\eta _{{\text{p}}}^{2}$ = 0.94. Follow-up analyses revealed that, across groups, participants rated themselves as feeling significantly more unpleasant after the mood induction than prior to the induction, *F*(1,37) = 205.57, *p* < 0.001, $\eta _{{\text{p}}}^{2}$ = 0.85, and that they felt significantly more pleasant after the memory cuing procedure, compared to prior to the induction, *F*(1,37) = 24.65, *p* < 0.001, $\eta _{{\text{p}}}^{2}$ = 0.40, and following the negative mood induction clip, *F*(1,38) = 484.26, *p* < .001, $\eta _{{\text{p}}}^{2}$ = 0.93. No main effect, *F*(1,37) = 1.14, *p* = 0.29, $\eta _{{\text{p}}}^{2}$ = 0.03, or interactions, *F*(1,37) = 0.18, *p* = 0.68, $\eta _{{\text{p}}}^{2}$ = 0.01, with Group were found.

Participants pleasantness ratings decreased after the induction and then increased again after memory cuing, irrespective of the personal or non-personal nature of the memory cue.

#### Arousal

The mixed ANOVA for arousal ratings showed a significant main effect of time, *F*(1,37) = 11.76, *p* ≤ . 001, $\eta _{{\text{p}}}^{2}$ = 0.24. The covariate variable had no effects on the subsequent results, *F*(1,37) = 0.59, *p* = 0.45, $\eta _{{\text{p}}}^{2}$ = 0.02. Follow-up analyses revealed that, across groups, participants rated themselves as feeling significantly more aroused after the mood induction than prior to the induction, *F*(1,38) = 71.66, *p* ≤ 0.001,$~\eta _{{\text{p}}}^{2}$ = 0.65, and that they felt significantly less aroused after the memory cuing procedure than prior to it, *F*(1,38) = 59.19, *p* ≤ 0.001, $\eta _{{\text{p}}}^{2}$ = 0.61. Finally, after cue presentation, participants’ levels of arousal returned to baseline and did not differ significantly from pre-induction levels, *F* (1,38) = 1.29, *p* = 0.26, $\eta _{{\text{p}}}^{2}$ = 0.03. No main effects for group or interaction of group by time were found, *F*(1,37) = 0.76, *p* = 0.39, $\eta _{{\text{p}}}^{2}$ = 0.02, *F*(1,37) = 0.10, *p* = 0.75, $\eta _{{\text{p}}}^{2}$ = 0.00, respectively.

Participants arousal ratings increased after the induction and then decreased again after memory cuing, irrespective of the personal or non-personal nature of the memory cue.

#### Dominance

The mixed ANOVA for dominance ratings showed a significant main effect of time, *F*(2,37) = 42.95, *p* < .001, $\eta _{{\text{p}}}^{2}$ = 0.69. Follow-up analyses revealed that, across groups, participants rated themselves as feeling significantly less dominant after the mood induction than prior to the induction, *F* (1,38) = 61.02, *p* < 0.001, $\eta _{{\text{p}}}^{2}$ = 0.62, and that they felt significantly more dominant after the memory cuing procedure than following the induction, *F*(1,38) = 73.30, *p* < 0.001, $\eta _{{\text{p}}}^{2}$ = 0.66. No significant differences in dominance between pre-induction and post-cuing were found, *F*(1,38) = 1.39, *p* = 0.24, $\eta _{{\text{p}}}^{2}$ = 0.04. No main effects, *F*(1,38) = 0.34, *p* = 0.56, $\eta _{{\text{p}}}^{2}$ = 0.01, or interactions, *F*(1,38) = 0.12, *p* = 0.74, $\eta _{{\text{p}}}^{2}$ = 0.00, with group were found.

Participants’ dominance ratings decreased after the induction and then increased again after memory cuing, irrespective of the personal or non-personal nature of the memory cue.

#### Nostalgia and reliving

Regarding scores on nostalgia and reliving generated by cues, no significant differences were found between groups for nostalgia (Personal: *M* = 1.42 and SD = 0.61; Non-personal: *M* = 1.19 and SD = 0.51) and sense of reliving (Personal: *M* = 1.16 and SD = 0.50; Non-personal: *M* = 1.43 and SD = 0.59). Participants shown non-personal images exhibited a significant positive correlation between the reliving sensation evoked by the images and how pleasant these images made them feel (*r* = .49, *p* = .023), whereas no significant correlation between these variables was evident for participants shown personal images (*r* = .26, *p* = .278). No significant correlations between reliving reports and feelings of arousal or dominance were evident for either group (all *ps* > 0.26). Regarding nostalgia ratings, no significant associations with pleasantness, dominance or arousal ratings were observed in either group (all *ps* > 0.39).

## Discussion

The present study investigated the possible differential effects of memories cued by personal or non-personal images on older adults’ abilities to regulate their mood after a negative mood induction. To this end, we induced a negative mood through presenting a scene from the film “Dead Man Walking” [16] and subsequently exposed participants to a number of positive images that they either supplied themselves or were selected from the IAPS set of generic images [17]. The retrieval of positive autobiographical memories has previously been found to aid in the repair of negative mood states [8, 22]. Direct cues, such as those which refer explicitly to an autobiographical event, are also presumed to aid in the retrieval of such positive memories compared to generative cues, such as generic imagery, where the self-relevance of such images must first be processed before a relevant memory can be retrieved. Given the difficulty that older adults have been found to have in accessing specific autobiographical memories it was hypothesised that greater mood-reparative effects would be found for the personal images, which should evoke direct retrieval, compared to the non-personal images, which should evoke generative retrieval.

First, regarding the negative emotion induction, after viewing the clip from “Dead Man Walking” [16] participants showed significantly diminished feelings of positivity, dominance and increased feelings of arousal compared to prior to the manipulation. This finding adds further support to the effectiveness of this clip in evoking a change in mood amongst older adults, as has been found elsewhere [23]. Furthermore, it is of note that no significant differences in mood were found between participants that viewed the entire clip and those that chose to stop the clip early. This clip appears to evoke an early shift in mood that persists whether or not the clip is watched until its end.

However, contrary to our hypotheses, both groups showed significantly improved mood after the memory cuing procedure and there was no difference in the extent of this recovery between participants who were given personal cues and those who were given non-personal cues. Participants in both groups may have been able to generate a positive autobiographical memory in response to the cues, regardless of whether the image was personal or not, and use this to repair their mood.

Despite the impersonal nature of the IAPS images used in this experiment, the group who were shown these images reported similar levels of nostalgia and reliving as participants who were shown personal images. Interestingly, for participants in the non-personal group, those participants who reported stronger reliving of their autobiographical memories after cuing also reported feeling more pleasant than those with weaker reliving. It seems then that the mood-reparative effects of IAPS images is influenced by the extent to which they evoke a sense of reliving in people, whereas for personal images this is not the case. These findings suggest that generic imagery can be used as a feasible and effective alternative to personal imagery in memory therapeutic interventions such as Life Review [3] where personal imagery may not be accessible or may not exist in enough quantity to evoke enough memories across a weeks-long intervention programme. However, it also suggests that clinicians should ensure that such generic imagery evokes a sense of reliving amongst participants to ensure that it enables participants to regulate their moods.

In the present study, the induction clip evoked an increase in arousal and the subsequent memory cuing procedure subsequently evoked a decrease in arousal. This suggests that arousal increases in older adults in response to unpleasant stimuli whilst decreasing in response to pleasant stimuli. This is similar to the findings reported by Grühn and Scheibe [24] and Keil and Freund [25], who used images from the IAPS [17] and found that higher negative valence ratings were associated with lower arousal scores and vice versa, and that this effect was particularly pronounced amongst older adults.

Some limitations of the present study are apparent. First, there was no measurement of the memories that participants retrieved therefore it is not possible to judge the positivity of these memories, their self-relevance or their degree of specificity. In the current study, only the feelings of relieving and nostalgia evoked by the images and their accompanying memories were explored. Our hypotheses were based on the understanding that personal images would allow for better, or more direct, access to specific autobiographical memories compared to the non-personal images which might be more likely to evoke the lengthier process of generative memory retrieval, However, given that there was no difference between groups in their reports of nostalgia and reliving it seems that both groups retrieved memories of equivalent self-relevance and vividness. Also, as both groups showed similar improvements in their mood it seems unlikely that there were group differences in the positivity of the memories that they retrieved. We also did not record the length of time that participants took to retrieve their memories. In the present design participants were given an unlimited amount of time to retrieve their memory, therefore both groups may have eventually retrieved a relevant specific memory and so have benefited from the mood-reparative effects of these memories. It may still be that personal images allow for more direct and immediate access to autobiographical memories, but such effects may only be evident if participants are limited in the time that they have to retrieve a positive memory or when they are being distracted by concurrent tasks they may be performing. Future studies should examine whether restrictions on retrieval time or differences in the retrieval environment elicit differential effects of personal, compared to non-personal, imagery on memory retrieval and emotion regulation.

In addition, our hypotheses were that personal cuing procedures would be particularly beneficial amongst older adults given the difficulty that they experience in retrieving specific autobiographical memories [12]. However, our study did not include a comparison with younger participants, therefore it may be that although our older participants showed mood-reparative effects, that this was reduced relative to that which have been observed amongst younger participants. Future replications of this investigation should have an additional comparison with younger participants.

Similarly, the absence of a control group who did not receive any cuing procedure means that we are unable to rule out whether the improvements in mood that were observed in the present study after the cuing procedure were fully attributable to the cuing procedure or whether it was attributable to mere regression to the mean. This latter conclusion seems unlikely to explain the observed effects given the reports of nostalgia and reliving in both groups and the correlation between reliving and feelings of positivity in the non-personal group. Nevertheless, future studies should include a non-cued control group.

Regarding sample size, post-hoc sensitivity analysis suggested that, given a total sample size of 39, this investigation could detect effects that were in the large range (e.g., partial eta-squared of 0.30) with 80% power. The novelty of the present investigation limited our ability to conduct an a priori power analysis and determine our sample size on this basis. Although our previous investigation in this area [6] did not examine the differential effects of personal vs. non-personal cues on recall or mood repair, it also included a total sample size of approximately 40 participants with 2 groups and observed changes in depressive moods following recall of positive autobiographical memories in the large range (partial eta-squared = 0.49). Our analysis replicated these effects in terms of improvements in mood after memory cuing (partial eta-squared = 0.40). However, there was limited evidence to suggest that personal vs. non-personal cues can have a differential impact on these mood-reparatory effects. Further to this, our sample showed a wide age range (65–92 years old) and in both groups the samples were predominantly made up of females. Due to our limited sample size, we were not able to perform analyses accounting for the possible effects of age and gender on our other variables of interest. These findings therefore warrant further replication in larger samples.

In conclusion, positive autobiographical memory cued by personal and non-personal images evoke feelings of nostalgia and reliving [11] and encourage mood-reparative effects during negative mood states [8]. Contrary to our hypotheses, despite the memory disruptions that older adults have previously shown [12], both personal and non-personal images elicited similar mood-reparatory effects after a negative mood induction. Our data suggest that a suitable selection of positive non-personal autobiographical memories can generate an associated memory with high levels of nostalgia and so be used to help older adults regulate their emotions as part of memory therapeutic interventions such as Life Review.

## Electronic supplementary material

Below is the link to the electronic supplementary material.

Supplementary material 1 (SAV 17 KB)

## References

[1] Tulving E (2002). Episodic memory: From mind to brain. Annu Rev Psychol.

[2] Nelson K, Fivush R (2004). The emergence of autobiographical memory: a social cultural developmental theory. Psychol Rev.

[3] Serrano JP, Latorre JM, Ros L (2012). Life review therapy using autobiographical retrieval practice for older adults with clinical depression. Psicothema.

[4] Williams JMG, Barnhofer T, Crane C (2007). Autobiographical memory specificity and emotional disorder. Psychol Bull.

[5] Conway MA, Pleydell-Pearce CW (2000). The construction of autobiographical memories in the self-memory system. Psychol Rev.

[6] Serrano JP, Latorre JM, Gatz M, Montañés J (2004). Life review therapy using autobiographical retrieval practice or older adults with depressive symptomatology. Psychol Ageing.

[7] Barclay CR, Smith TS, Conway MA, Rubin DC, Spinnler H, Wagenaar WA (1992). Autobiographical remembering: creating personal culture. Theoretical perspectives on autobiographical memory.

[8] Joormann J, Siemer M (2004). Memory accessibility, mood regulation, and dysphoria: difficulties in repairing sad mood with happy memories?. J Abnorm Psychol.

[9] Joormann J, Siemer M, Gotlib IH (2007). Mood regulation in depression: differential effects of distraction and recall of happy memories on sad mood. J Abnorm Psychol.

[10] Werner-Seidler A, Moulds ML (2012). Mood repair and processing mode in depression. Emotion.

[11] El-Ziab N (2016). Walking down memory lane: the unfolding experience of nostalgia. Psychol Soc.

[12] Wilson FCL, Gregory JD (2018). Overgeneral autobiographical memory and depression in older adults: a systematic review. Aging Ment Health.

[13] Addis DR, Knapp K, Roberts RP, Schacter DL (2012). Routes to the past: neural substrates of direct and generative autobiographical memory retrieval. NeuroImage.

[14] Uzer T, Brown NR (2017). The effect of cue content on retrieval from autobiographical memory. Acta Physiol (Oxf).

[15] Watt L, Cappeliez P (2000). Integrative and instrumental reminiscence therapies for depression in older adults: Intervention strategies and treatment effectiveness. Ageing Mental Health.

[16] Robbins T, Kilik J, Simmon R, Robbins T (1995). Dead man walking [Cinta Cinematográfica].

[17] Lang PJ, Bradley MM, Cuthbert BN (2008) International affective picture system (IAPS): affective ratings of pictures and instruction manual. Technical Report A-8. University of Florida, Gainesville, FL

[18] Cella D, Riley W, Stone A, Rothrock N, Reeve B, Yount S, Hays R (2010). The patient-reported outcomes measurement information system (PROMIS) developed and tested its first wave of adult self-reported health outcome item banks: 2005–2008. J Clin Epidemiol.

[19] Watson D, Clark LA, Tellegen A (1988). Development and validation of brief measures of positive and negative affect: the PANAS Scales. J Pers Soc Psychol.

[20] Fernández CF, Mateos JCP, Ribaudi JS, Fernández-Abascal EG (2011). Validación española de una batería de películas para inducir emociones. Psicothema.

[21] Lang PJ, Sidowski JB, Johnson JH, Williams TA (1980). Behavioral treatment and bio-behavioral assessment: computer applications. Technology in mental health care delivery systems.

[22] Smith SM, Petty RE (1995). Personality moderators of mood congruency effects on cognition: the role of self-esteem and negative mood regulation. J Person Soc Psychol.

[23] Fernández-Aguilar L, Ricarte J, Ros L, Latorre JM (2018). Emotional differences in young and older adults: films as mood induction procedure. Front Psychol.

[24] Grühn D, Scheibe S (2008). Age-related differences in valence and arousal ratings of pictures from de International affective picture system (IAPS): do ratings become more extreme with age?. Behav Res Methods.

[25] Keil A, Freund AM (2009). Changes in the sensitivity to appetitive and aversive arousal across adulthood. Psychol Aging.

